# ChEMBL-Likeness Score and Database GDBChEMBL

**DOI:** 10.3389/fchem.2020.00046

**Published:** 2020-02-04

**Authors:** Sven Bühlmann, Jean-Louis Reymond

**Affiliations:** Department of Chemistry and Biochemistry, University of Bern, Bern, Switzerland

**Keywords:** chemical space exploration, molecular database, enumeration algorithm, chemical space mapping, virtual screening

## Abstract

The generated database GDB17 enumerates 166.4 billion molecules up to 17 atoms of C, N, O, S and halogens following simple rules of chemical stability and synthetic feasibility. However, most molecules in GDB17 are too complex to be considered for chemical synthesis. To address this limitation, we report GDBChEMBL as a subset of GDB17 featuring 10 million molecules selected according to a ChEMBL-likeness score (CLscore) calculated from the frequency of occurrence of circular substructures in ChEMBL, followed by uniform sampling across molecular size, stereocenters and heteroatoms. Compared to the previously reported subsets FDB17 and GDBMedChem selected from GDB17 by fragment-likeness, respectively, medicinal chemistry criteria, our new subset features molecules with higher synthetic accessibility and possibly bioactivity yet retains a broad and continuous coverage of chemical space typical of the entire GDB17. GDBChEMBL is accessible at http://gdb.unibe.ch for download and for browsing using an interactive chemical space map at http://faerun.gdb.tools.

## Introduction

Innovation at the level of chemical structures is an essential part of drug discovery. Novelty often results from chemical intuition however this approach is increasingly difficult as the number of known molecules increases. Novelty is similarly limited in virtual combinatorial libraries (Leach and Hann, 2000; Hu et al., 2011; van Hilten et al., 2019) and generative models trained with known molecules (Chen et al., 2018; Elton et al., 2019) because these systems mostly shuffle known patterns, which produces many technically new but often not fundamentally innovative molecules. To circumvent this limitation, we have initiated the exhaustive enumeration of all possible organic molecules following simple rules of chemical stability and synthetic feasibility, and reported large databases enumerating molecules up to 11 (Fink et al., 2005; Fink and Reymond, 2007), 13 (Blum and Reymond, 2009), and 17 atoms (Ruddigkeit et al., 2012, 2013), as well as of possible ring systems up to 30 atoms (Visini et al., 2017a). Analyzing the resulting generated databases (GDBs) shows that there are many orders of magnitude more possible molecules spanning a much broader structural diversity than already known ones (Reymond, 2015; Awale et al., 2017b).

One of the defining features of the GDB databases is the exponential increase in the number of possible molecules as function of increasing molecular size and complexity elements, such as stereocenters and heteroatoms, implying that most possible molecules are in fact far too complex to be considered as realistic synthetic targets. To address this problem we have designed subsets of our largest database GDB17 by limiting complexity elements using simplification criteria, such as fragment-likeness (Congreve et al., 2003), producing the fragment database FDB17, and medicinal chemistry rules for functional groups and complexity (Mignani et al., 2018), producing the medicinal chemistry aware database GDBMedChem (Visini et al., 2017b; Awale et al., 2019). These approaches however also constrain the diversity of GDB molecules, which partly defeats the purpose of exploring chemical space broadly.

**Figure 1:**
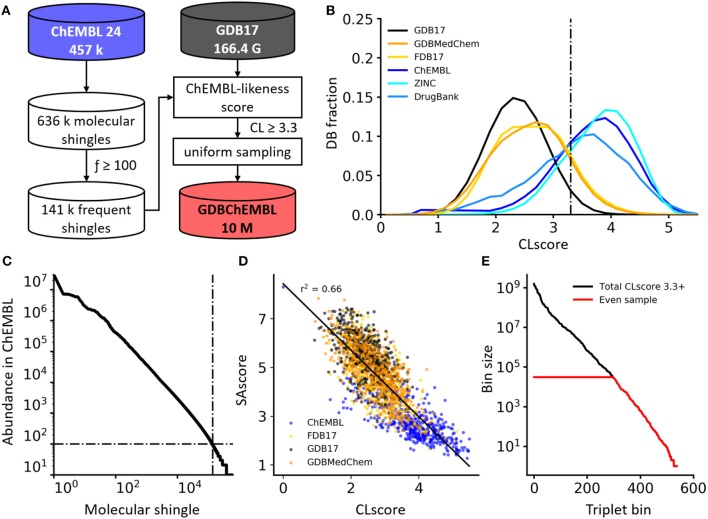
**(A)** Generation process of GDBChEMBL. **(B)** CLscore distributions for GDB17, its subsets FDB17 and GDBMedChem, and public databases ChEMBL, ZINC, and DrugBank. **(C)** Frequency distribution of molecular shingles up to a diameter of 6 bonds in ChEMBL. **(D)** SAscore vs. CLscore in various databases. A lower SAscore indicates higher synthetic accessibility, and a higher CLscore indicates higher similarity to ChEMBL molecules. **(E)** Occupancy of triplet value bins (HAC, stereocenters, heteroatoms) in all GDB17 cpds with CLscore ≥3.3 (black line) and after uniform sampling forming GDBChEMBL (red line).

Herein we report an alternative approach to create subsets of GDB17 based on the frequency of occurrence of substructures from known molecules independent of the overall molecular structure (Figure 1A). We define a “ChEMBL-likeness” score (CLscore) by considering which substructures in a molecule also occur in molecules from the public database ChEMBL (Gaulton et al., 2017), using a subset of molecules with reported high confidence datapoint of activity on single protein targets, a type of ChEMBL subset which we have used previously for target prediction (Awale and Reymond, 2019; Poirier et al., 2019). We then filter the entire GDB17 with a cut-off value for CLscore, followed by uniform sampling of the resulting subset across molecular size, stereocenters and heteroatoms as done previously with FDB17 and GDBMedChem, to obtain a ChEMBL-like subset of 10 million molecules forming the database GDBChEMBL. This database covers chemical space as broadly as but more continuously than FDB17 and GDBMedChem yet features a much higher synthetic accessibility as judged by a calculated synthetic accessibility score (Ertl and Schuffenhauer, 2009), might contain molecules with a higher probability of bioactivity, and in any case provides a very different starting point to serve as a source of inspiration for molecular design.

## Results and Discussion

### ChEMBL-Likeness Score

Our definition of CLscore is related to the synthetic accessibility score (SAscore) (Ertl and Schuffenhauer, 2009) and natural product likeness score (NPscore) (Jayaseelan et al., 2012) of a molecule, which are calculated from the occurrence frequencies of its substructures in PubChem and fragments from natural products, respectively, combined with additional functional group rules. Here we focus on 457,139 compounds recorded in ChEMBL24 as being active on single protein targets (IC_50_ or EC_50_ ≤ 10 μM) with high confidence datapoints (Awale and Reymond, 2019; Poirier et al., 2019). To design our CLscore we consider circular substructures, called molecular shingles, because they form the basis for molecular fingerprints ECFP4 and MHFP6 which perform best in benchmarking studies (Riniker and Landrum, 2013; Probst and Reymond, 2018).

The frequency of occurrence of the 636,979 molecular shingles up to a diameter of six bonds found in our ChEMBL subset follows a power law distribution (Figure 1C). To compute the CLscore of a molecule, we assign to each of its shingles (S) a shingle value calculated from the logarithm of its frequency of occurrence f_S_ in our ChEMBL subset, considering only shingles occurring at least 100 times in this subset (141,261 shingles, 22.2% of the total). We then sum all shingle values and divide the sum by the total number of shingles in the molecule (Equation 1).

$$\begin{array}{l} \text{CLscore}=\frac{\sum_{\text{i}=1}^{\text{m}}{\text{log}}_{10}{\left(f_{\text{S}}\right)}_{\text{i}}}{\text{N}}|\begin{array}{l} \text{CLscore}:=\text{ChEMBL}-\text{likeness score}\\ \text{S}:=\text{shingleinmolecular structure}\\ f_{\text{S}}:=\text{abundanceofmolecularshinglein ChEMBL}\\ \text{N}:=\text{totalnumberofshinglesinmolecular structure}\\ \text{m}:=\text{numberofshinglessharedwith ChEMBL} \end{array} \end{array}$$

The histogram of CLscore for the 457,139 ChEMBL reference molecules is approximately Gaussian with a peak at CLscore = 3.9 (Figure 1B). DrugBank (Law et al., 2014) and particularly ZINC (Sterling and Irwin, 2015) peak at a similar CLscore, showing that these three databases consist of molecules built from the same type of substructures. By contrast GDB17 and its subsets FDB17 and GDBMedChem have a much lower CLscore distribution peaking at CLscore = 2.7, reflecting the fact that GDB molecules are very different from ChEMBL molecules. CLscore values correlate with SAscore values, reflecting the similar principles underlying both scores, and suggesting that molecules with high CLscore should also be synthetically accessible (Figure 1D).

### GDBChEMBL Database

Calculating CLscores on the entire GDB17 (166.4 billion SMILES) and keeping molecules with CLscore ≥ 3.3, a cut-off value which retains 78.3% of our ChEMBL subset, eliminates 84.3% of GDB17. The remaining 26.2 billion molecules are then binned in triplet value bins considering heavy atom count (HAC 1-17), stereocenter count (0–4, ≥ 5) and heteroatom count (0–8, ≥ 8). There are 538 different triplet value bins, which are occupied by 1 to 1.6 × 10^9^ molecules. Uniform sampling finally yields a final set of 10 million molecules evenly distributed across molecular size, stereochemical complexity and polarity, forming the database GDBChEMBL (Figure 1E).

**Figure 2:**
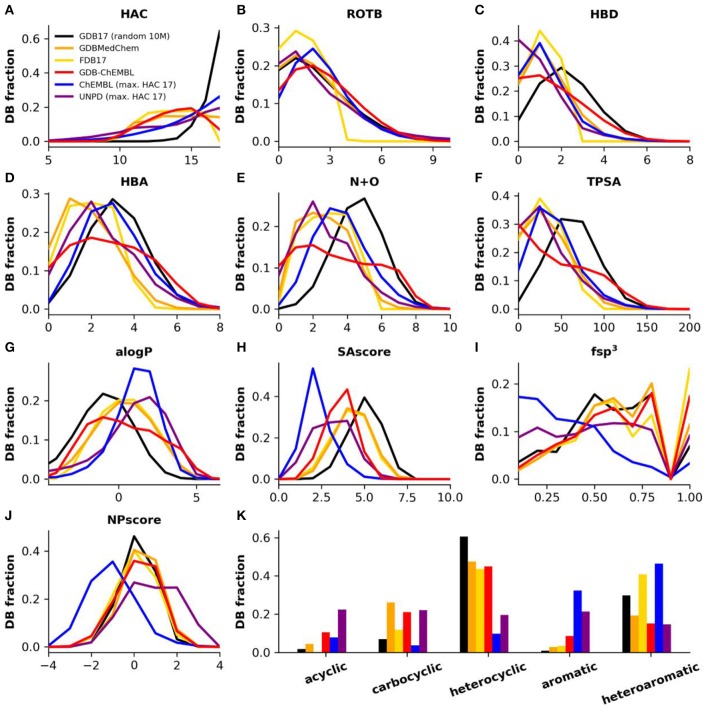
**(A)** Heavy atom count (HAC), **(B)** rotatable bonds (ROTB), **(C)** hydrogen bond donors (HBD), **(D)** hydrogen bond acceptors (HBA), **(E)** nitrogen plus oxygen count (N+O), **(F)** topological polar surface area (TPSA), **(G)** computed partition coefficient (aLogP), **(H)** synthetic accessibility score (SAscore), **(I)** Fraction of sp^3^ hybridized atoms (fsp^3^), **(J)** natural product likeness score (NPscore), and **(K)** fraction of structures by ring class. Property histograms for GDB17 (black), GDBMedChem (orange), FDB17 (yellow), GDBChEMBL (red), ChEMBL (cpds with HAC ≤ 17, blue) and natural products (cpds with HAC ≤ 17, purple).

As a consequence of uniform sampling, the heavy atom count (HAC) profile of GDBChEMBL resembles that of FDB17 and GDBMedChem and is relatively flat compared to the very steep peak at HAC = 17 in the parent database GDB17 (Figure 2A). Uniform sampling also explains the rotatable bond count (RBC) profile in GDB subsets compared to GDB17 (Figure 2B), as well as the fact that the profiles of the three GDB subsets across these parameters are generally more similar to the profile of molecules up to 17 atoms in ChEMBL (ChEMBL17) and to natural products (UNPD17) (Banerjee et al., 2015) than to the profile of GDB17.

GDBChEMBL displays a very broad distribution in terms of hydrogen bond donor atoms (HBD, Figure 2C), hydrogen bond acceptor atoms (HBA, Figure 2D) and nitrogen plus oxygen atom count (N+O, Figure 2E) due to the absence of heteroatom capping criteria in selecting GDBChEMBL compared to FDB17 and GDBMedChem, for which fragment-likeness criteria, respectively, caps on the number of functional groups were applied. Similar differences are visible in topological polar surface area (TPSA, Figure 2F) and calculated octanol/water partition coefficient (alogP, Figure 2G). The broader distribution of polarity parameters in GDGChEMBL compared to GDB17 results from uniform sampling since the procedure gives relatively more importance to molecules with extreme size and polarity values.

Synthetic accessibility is better (lower SAscore) in GDBChEMBL than for GDB17, FDB17, or GDBMedChem, reflecting the correlation between CLscore and SAscore noted above (Figure 2H). Similar to GDB17 and its other subsets, GDBChEMBL displays a much higher fraction of sp^3^ atoms than ChEMBL (fsp^3^, Figure 2I). As a consequence GDB molecules are closer to natural products, which is reflected in the NPscore profile (Figure 2J). Despite of these differences and similarities in SAscore and NPscore, it must be noted that GDB17 and its subsets stand out by the fact that they contain fewer aromatic and more heterocyclic molecules than ChEMBL and natural products (Figure 2K).

### Visualization and Similarity Searching

To gain an overview of GDBChEMBL we computed Molecular Quantum Number (MQN) fingerprint values (Nguyen et al., 2009), performed a principal component analysis (Rosén et al., 2009), and visualized the resulting 3D-map in the interactive web-based application faerun (Probst et al., 2018). In this 3D-map accessible at http://faerun.gdb.tools, each point represents one or more molecules present at the corresponding position and can be color-coded according to a molecular property selected from the faerun menu.

**Figure 3:**
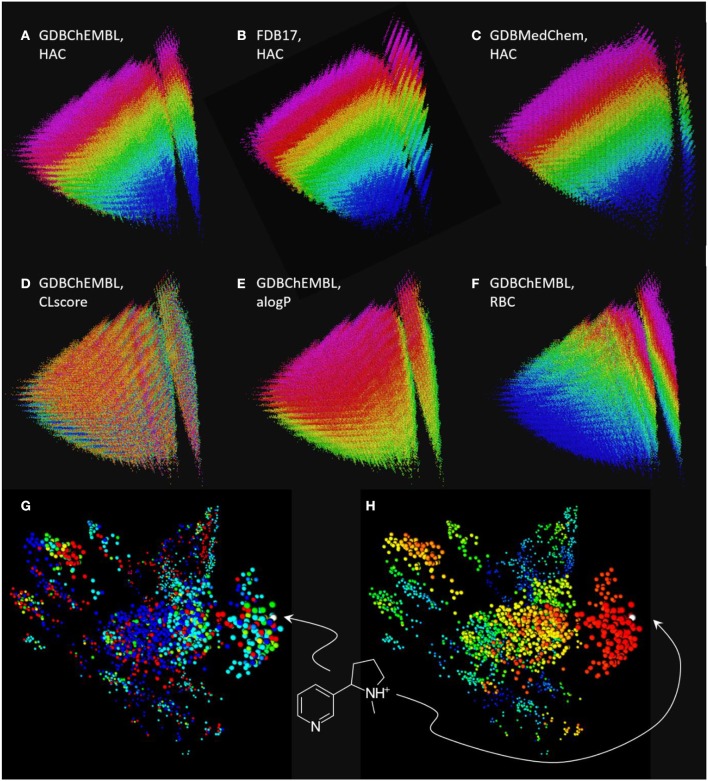
Chemical space maps of GDBChEMBL, FDB17, and GDBMedChem. **(A)** PCA 3D-map of GDBChEMBL in MQN-space, color coded by heavy atom count; **(B)** same as a for FDB17; **(C)** same as a for GDBMedChem; **(D)** GDBChEMBL color-coded by CLscore value; **(E)** GDBChEMBL color-coded by calculated octanol/water partition coefficient alogP; **(F)** GDBChEMBL color-coded by rotatable bond count; **(G)** similarity map of MQN-nearest neighbors of nicotine from GDBChEMBL (red), FDB17 (cyan), and GDBMedChem (blue). Points in green and yellow indicate molecules shared by two databases. **(H)** Same as g color-coded by Xfp-similarity to nicotine. MQN maps a to f are accessible at http://faerun.gdb.tools. The similarity map of nicotine analogs g and h is accessible at: http://gdbtools.unibe.ch:8080/webMolCS/.

Comparing MQN maps of GDBChEMBL, FDB17 and GDBMedChem shows that each of the three GDB17 subset cover a similar range of properties, however coverage by GDBChEMBL is more continuous, as is well visible in the vertical stripe at right containing all acyclic molecules (Figures 3A–C). Note that CLscore values are not correlated with MQN properties, which is not surprising considering that ChEMBL substructure span a broad range of properties (Figure 3D). Color-coding by the calculated logP value (alogP, Figure 3E) and by rotatable bond count (RBC, Figure 3F) illustrate the distribution of molecules in the MQN map.

The fact that molecules in GDBChEMBL are substantially different from those in the other subsets FDB17 and GDBMedChem can be shown by retrieving 1,000 MQN-nearest neighbors of nicotine from each database, and representing each dataset in a similarity map (Medina-Franco et al., 2007; Raghavendra and Maggiora, 2007; Awale and Reymond, 2015) using the molecular shape and pharmacophore fingerprint Xfp (Awale and Reymond, 2014), computed with the web-based application WebMolCS (Awale et al., 2017a). This visualization shows that each database provides different types of nicotine analogs (Figure 3G) with a good number of high similarity analogs (Figure 3H). To facilitate similarity searches in GDBChEMBL, we have implemented a similarity search portal by which nearest neighbor searches of any molecule can be performed in GDBChEMBL using MQN, ECFP4, or a combined MQN-MHFP6 similarity, as described previously for GDBMedChem (Awale et al., 2019).

## Conclusion

The data above demonstrate a substructure-based approach to select molecules from the generated database GDB17. As selection criterion we defined a ChEMBL-likeness score (CLscore) from the frequency occurrence of circular substructures, called molecular shingles, in a subset of the database ChEMBL consisting of compounds active on single protein targets with high confidence datapoints. This selection reduced GDB17 by 84.3%, leaving 26.2 billion molecules, which we sampled uniformly across molecular size, stereochemistry and heteroatoms to form GDBChEMBL comprising 10 million molecules.

Property profiles, chemical space maps and similarity searches show that GDBChEMBL is very different from our earlier GDB subsets FDB17 and GDBMedChem and spans chemical space more continuously. At the same time, the correlation between CLscore and the synthetic accessibility score (SAscore) implies that GDBChEMBL molecules will be on average easier to synthesize than molecules from FDB17 and GDBMedChem, which have significantly lower CLscore and higher SAscores. We anticipate that the requirements for GDBChEMBL molecules to share a minimum number of substructures with molecules of known bioactivities from ChEMBL will also facilitate target prediction and the selection of interesting GDB molecules for synthesis and testing.

## Methods

### Preparative Steps

#### ChEMBL Shingle Extraction

The ChEMBL (v 24.1) database was downloaded from https://www.ebi.ac.uk/chembl/. Data points for extraction of molecular shingles were selected by applying the same restrictions that were used for extraction of training data for our Polypharmacology Browser PPB2 (Visini et al., 2017b). Structures were normalized to their major protonation state at pH 7.4 using ChemAxon cxcalc (v. 18.23.0). Molecular shingles for radii 1–3 were created using RDkit (2019.03.4) and converted to rooted, canonical, aromatic SMILES strings without retaining stereochemistry information. In association with abundancy in the ChEMBL, the SMILES substructures were stored as pickled python dictionary. Molecular substructures that were found <100 times were not stored.

#### CLscore Calculation

Scoring of GDB17 molecular structures was achieved by decomposition to molecular shingles in the exact same way as described for ChEMBL reference shingle extraction. For a specific query structure, all shingles are uniquely counted, then looked up in the ChEMBL reference database and upon match, logarithmic abundancy is summed up. The final CLscore is given by the ratio of total logarithmic abundancies of matched unique shingles to total unique shingles in the query structure. All respective scripts are accessible at: https://github.com/reymond-group/GDBChEMBL.

#### GDBChEMBL Generation

All 166.4 billion molecular structures of GDB17 were decomposed to unique substructures in the same way as described for ChEMBL reference molecules. Only structures with CLscore ≥3.3 were stored. The final GDBChEMBL was obtained by distribution of all filtered 26.2 billion structures to 538 property triplet bins (heavy atom, heteroatom and stereocenter count). Property information was gathered using RDKit. Bins with 5+ hetero atoms and/or 8+ chiral atoms were merged. The actual even sampling was performed by sorting all property bins by size and defining target structure count as 10 million. Iteratively, remaining target count was divided by count of remaining bins, keeping all bins of size smaller than the current number to sample randomly. For each step, number of previously selected structures was subtracted from target count until random sample per remaining bins was lower than bin size. At this point, sample size was kept constant for all further bins.

#### Visualizing GDBChEMBL in Faerun

Property color coded 3D maps for GDBChEMBL, FDB17, and GDBMedChem were generated using FUn (doc.gdb.tools/fun), an in-house developed framework for interactive visualization of chemical spaces on the web. Datasets were given as plain text, consisting of the four columns (space-separated): SMILES-string, numeric ID, 42 MQN descriptors (semicolon-separated) and further molecular properties used for map coloring (semicolon-separated). Next, the preprocessing toolchain was used to project the 42-dimensional MQN-space to 3D by applying Principal Component Analysis (PCA) and to generate all further files needed for visualization. Finally, the Underdark server was run using docker with Faerun visualization containers mapped.

#### Similarity Searching in GDBChEMBL

For better accessibility, GDBChEMBL is provided as a web-based interactive similarity search tool. The implementation uses HTML, Bootstrap, JavaScript, and the python Flask framework. Search times were reduced using Annoy trees (Approximate Nearest Neighbors Oh Yeah, https://github.com/spotify/annoy) which were created for the 42-dimensional MQN property space, as well as for 256-bit ECfp4. A third search option, MQN-MHFP6, initially searches using the MQN Annoy tree followed by resorting after Jaccard distance to query molecule in the MHFP6 fingerprint space (https://github.com/reymond-group/mhfp). The search tool is available at: gdb.unibe.ch/tools.

## Data Availability Statement

All datasets generated for this study are included in the article/supplementary material.

## Author Contributions

SB designed and realized the study and wrote the paper. J-LR designed and supervised the study and wrote the paper.

### Conflict of Interest

The authors declare that the research was conducted in the absence of any commercial or financial relationships that could be construed as a potential conflict of interest.
